# Exploring perceptual disparities: A study on the level of understanding of colorectal cancer care among patients and healthcare professionals

**DOI:** 10.1007/s00384-025-05064-9

**Published:** 2026-01-08

**Authors:** Eleftherios Christodoulis, Panagiotis Ntellas, Lilly Simpson, Katerina Dadouli, Jacqueline Connell, Kok Haw Jonathan Lim, Joseph Williams, Jurjees Hasan, Marios Adamou, Saifee Mullamitha, Daniel Anderson, Francisca Marti Marti, Michael Braun, Mark Saunders, Tess Gillham, Konstantinos Kamposioras

**Affiliations:** 1 Department of Medical Oncology, The Christie NHS Foundation Trust, Manchester, M20 4BX UK; 2 Department of Medical Oncology, The Royal Marsden Hospital, Sutton, SM2 5 UK; 3 Laboratory of Hygiene and Epidemiology, Faculty of Medicine, University of Thessaly, 41222 Larissa, Greece; 4 Division of Cancer Sciences, Faculty of Biology, Medicine and Health, The University of Manchester, Manchester, UK; 5 Advanced Immunotherapy and Cell Therapy Team, The Christie NHS Foundation Trust, Manchester, UK; 6 Division of Pharmacy and Optometry, School of Health Sciences, The University of Manchester, Manchester, M13 9 UK; 7 School of Human and Health Sciences, University of Huddersfield, Queensgate, Huddersfield HD1 3DH UK; 8 Department of Psycho-Oncology, The Christie NHS Foundation Trust, Manchester, M20 4BX UK; 9 Department of Clinical Oncology, The Christie NHS Foundation Trust, Manchester, M20 4BX UK

**Keywords:** Colorectal cancer, Perception, Concordance, Health care professional, Communication, Palliative care

## Abstract

**Background:**

Emotional engagement, family support and personal beliefs can influence how patients and healthcare professionals (HCPs) perceive cancer differently. This study examined the extent to which the views of patients and HCPs on cancer care align, and identified factors that may underlie disparities.

**Methods:**

Participants with colorectal cancer (CRC) were asked to describe their perception of their disease (i.e. whether they felt it was under control (DC), was progressing (PD), or was of an unknown status) and to complete psychometric assessments of anxiety, depression, PTSD and well-being. Two HCPs, who were blinded to the patients’ responses, examined the case files to determine the stage of treatment at which the patients were enrolled in the study. The concordance of perceptions between patients and HCPs was examined, along with associations with clinical variables and psychometric health outcomes, using both univariate and multivariate analyses.

**Results:**

A total of 205 patients with CRC were included in the study. The mean age was 65 years, with 58% of patients being male. Overall, a significant difference in perception was observed between HCPs and patients (p < 0.001), particularly for patients identified by HCPs as having PD. Significant discrepancies were observed among patients receiving palliative care (p < 0.001), whereas those in the adjuvant or neo-adjuvant pathway appeared to align more closely with HCPs’ perceptions (p = 0.99). Neither demographic nor psychological factors were significant determinants of concordance between HCPs and patients’ understanding of cancer status in this population. In multivariate analysis, patients perceiving PD or expressing uncertainty were found to have significantly higher levels of depression than those with DC (OR 6.42, p = 0.001 and OR 3.86, p = 0.009, respectively).

**Conclusions:**

This study reveals significant differences in how cancer is perceived by HCPs and patients, particularly among those without disease control or undergoing palliative care. This highlights the importance of effective communication in addressing patients’ needs and their psychological well-being.

**Supplementary Information:**

The online version contains supplementary material available at 10.1007/s00384-025-05064-9.

## Introduction

Cancer care requires close collaboration between patients and their healthcare professionals (HCPs), yet, their respective perceptions of the disease, treatment goals, and prognosis often diverge. Previous studies have shown that patients with cancer often struggle to fully understand the complex medical information and recommendations they receive [1–5]. These discrepancies can arise from a multitude of inter-related factors, including the patient’s background education, health literacy, lived experiences, family involvement, personal beliefs and, importantly, the intense emotional burden linked to the information being shared [5–7]. Processing medical information can be especially challenging for patients as they contend with fears about the future, worries for their family, and concerns about how cancer will impact their daily lives. This heightened emotional context can mediate and sometimes limit their ability to fully comprehend their diagnosis, prognosis, and treatment options, often requiring HCPs to revisit and clarify information over time to ensure understanding [6, 8, 9].

Similarly, the communication styles of HCPs can have profound effects on patient experiences and treatment choices [10]. Previous research suggests that HCPs may contribute to the creation of gaps in the patient’s understanding by focusing their language on the technical aspects of diagnosis, staging and treatment [3, 8]. In addition, some HCPs may feel reluctant to share negative news, leading to false perceptions of positive outcomes 10. It has also been suggested that HCPs may mistake a patient’s use of medical terminology for a signal that they understand their medical status, whereas some patients may adopt medical terms without fully understanding them, leading to underlying confusion [5, 6].

Misalignment in patients’ understanding of their care can negatively affect overall satisfaction and hinder their ability to make informed and meaningful choices about their treatment. Here, we set out to explore the extent of these discrepancies in perceptions between patients with colorectal cancer and their respective HCPs, and its impact on their overall cancer care experience.

## Materials and methods

### Study design and participants

The PICO-SM, a single-centre prospective survey study assessed the psychological impact of the COVID-19 pandemic on patients with colorectal cancer attending the specialist lower gastrointestinal cancer clinics at The Christie NHS Foundation Trust (Manchester, UK) [11, 12]. In brief, psychometric assessment was performed using validated self-reported tools: the Generalized Anxiety Disorder scale (GAD-7) for anxiety, the Patient Health Questionnaire-9 (PHQ-9) for depression, the Primary Care Post-Traumatic Stress Disorder-5 (PC-PTSD-5) for probable PTSD, and the World Health Organization Well-being Index (WHO-5) for overall mental well-being [13–16]. As previously described, patients identified through clinic lists were asked to indicate their understanding of their cancer status at the time of the survey [11]. Based on the responses, three discrete disease status groups could be defined: disease control (DC), progressive disease (PD) or uncertain (Table 1).

**Table 1:** Self-reported perception of current status of cancer

**Disease Control** **(DC)**
(Shrinking/responding well to treatment, under control or stable, have finished treatment & have routine monitoring scans/check-ups, have had curative treatment & no active cancer)
**Progressive disease (PD)**
(Progressing/getting worse)
**Uncertain**
(Recently diagnosed & waiting for treatment to start, undergoing investigations at this stage, don’t know or not certain, Other, *Prefer not to say)*

Two independent HCPs retrospectively reviewed the clinical records of patients enrolled in the PICO-SM study. They took into consideration the most recent imaging studies, tumour marker (CEA) trends, and the latest documented communications on electronic records, all before patients completed the survey. The aim was to assess indicators of disease status. Based on these parameters, the HCPs categorised the clinical status as DC, PD or uncertain. In the event of any disagreement between the two HCPs, a third, more senior HCP would provide an independent review, after which there would be a discussion to reach a consensus. The HCPs were blinded to the patients’ responses. Information was also collected on the intention of treatment, i.e. whether it was adjuvant, neoadjuvant or palliative.

### Study outcomes

This analysis aims to explore the extent to which patients’ perceptions of their cancer align with HCPs’ understanding, thereby highlighting the effectiveness of communication and mutual understanding between the two parties. The primary objective of the study was to assess the level of agreement or disagreement between patients’ perceptions of their cancer status and the views held by their healthcare providers. A secondary objective was to identify factors that may influence patients’ perceptions of their disease status, such as the intent of treatment (e.g., curative or palliative), and to understand how patients’ interpretations of their disease status impact their overall well-being.

### Statistical analysis

Statistical analyses were carried out using R language (R Core Team: R: A Language and Environment for Statistical Computing Vienna, Austria: Foundation for Statistical Computing. Available from: https://www.r-project.org/). Continuous variables were expressed as mean ± standard deviation and as median with interquartile range (IQR), and categorical variables were expressed as frequencies (n) and percentages (%). Categorical data were analysed using Chi-square test or Fisher’s exact test. Continuous variables were checked for deviation from normal distribution (Kolmogorov–Smirnoff normality test) for each comparison. Student’s t-Test or Mann–Whitney U Test were performed for continuous data, as appropriate. Multivariate analysis was performed using a logistic regression for binary outcomes (anxiety (GAD-7 score ≥ 5), depression (PHQ-9 score ≥ 10), and poor well-being (WHO-5 < 50)), and odds ratios (OR) with 95% confidence intervals (CIs) were calculated. For all the analyses, a 5% significance level was set, and p values were two-tailed.

## Results

### Study participants

A total of 205 participants, predominantly men (58.0%, n = 119/205), were enrolled in the study in April 2021 (Table 2). The mean age of the participants was 65 years (range 32 to 87 years), with the majority being White/White British (91.7%, n = 188/205). Majority (67.8%, n = 139/205) were in a relationship, married, or in a civil partnership, while 31.2% (n = 64/205) were single, divorced, separated, or widowed. Most participants (78.5%, n = 161/205) had children, and a significant majority (77.6%, n = 159/205) did not live alone (Table 2).

**Table 2:** Demographic characteristics

	Adjuvant or Neoadjuvant treatment (N = 43)	Palliative treatment (N = 162)	Overall (N = 205)
Gender			
Prefer not to say	0 (0%)	0 (0%)	0 (0%)
Male	25 (58.1%)	94 (58.0%)	119 (58.0%)
Female	17 (39.5%)	66 (40.7%)	83 (40.5%)
Other	0 (0%)	0 (0%)	0 (0%)
Missing	1 (2.3%)	2 (1.2%)	3 (1.5%)
Age			
Mean (SD)	65.0 (9.55)	65.5 (10.5)	65.4 (10.3)
Median [Min, Max]	66.0 [36.0, 81.0]	67.0 [32.0, 87.0]	67.0 [32.0, 87.0]
Ethnicity			
Prefer not to say	0 (0%)	0 (0%)	0 (0%)
White/White British	39 (90.7%)	149 (92.0%)	188 (91.7%)
Other	4 (9.3%)	11 (6.8%)	15 (7.3%)
Missing	0 (0%)	2 (1.2%)	2 (1.0%)
Marital status			
Prefer not to say	0 (0%)	0 (0%)	0 (0%)
Single/Divorced/Separated/Widowed	15 (34.9%)	49 (30.2%)	64 (31.2%)
In a relationship/Married/In civil partnership	28 (65.1%)	111 (68.5%)	139 (67.8%)
Missing	0 (0%)	2 (1.2%)	2 (1.0%)
Do you have children?			
No	10 (23.3%)	34 (21.0%)	44 (21.5%)
Yes	33 (76.7%)	128 (79.0%)	161 (78.5%)
Living Conditions			
With Company	32 (74.4%)	127 (78.4%)	159 (77.6%)
Alone	11 (25.6%)	34 (21.0%)	45 (22.0%)
Missing	0 (0%)	1 (0.6%)	1 (0.5%)

### Psychological health outcomes

The psychological outcomes for mental well-being, anxiety, depression and post-traumatic stress are presented in Table 3. A favourable mental well-being status was reported by 67.3% (n = 138/205) of the participants, according to the WHO-5 screening tool. Meanwhile, almost one in four participants (23.4%, n = 48/205) reported signs of anxiety. A total of n = 30/205 (14.6%) participants highlighted risk of depression, and a n = 5/205 (2.4%) participants reported possibility of the risk of significant post traumatic disorder.

**Table 3:** Psychological Health Outcomes

	Adjuvant or Neoadjuvant treatment (*N* = 43)	Palliative treatment (*N* = 162)	Overall (*N* = 205)
WHO-5 score			
Mean (SD)	65.8 (20.6)	57.1 (23.0)	58.9 (22.8)
Median [Min, Max]	72.0 [0, 100]	60.0 [0, 100]	64.0 [0, 100]
Well-Being			
Good (WHO-5 ≥ 50)	35 (81.4%)	103 (63.6%)	138 (67.3%)
Poor (WHO-5 < 50)	8 (18.6%)	59 (36.4%)	67 (32.7%)
GAD-7 score			
Mean (SD)	3.22 (5.66)	3.50 (4.83)	3.44 (5.00)
Median [Min, Max]	0 [0, 21.0]	1.00 [0, 20.0]	1.00 [0, 21.0]
Missing	2 (4.7%)	11 (6.8%)	13 (6.3%)
Anxiety			
No (GAD-7 < 5)	33 (76.7%)	111 (68.5%)	144 (70.2%)
Yes (GAD-7 ≥ 5)	8 (18.6%)	40 (24.7%)	48 (23.4%)
PHQ-9 score			
Mean (SD)	4.17 (5.35)	5.05 (5.22)	4.86 (5.24)
Median [Min, Max]	2.00 [0, 25.0]	4.00 [0, 23.0]	3.00 [0, 25.0]
Missing	1 (2.3%)	10 (6.2%)	11 (5.4%)
Depression			
No (PHQ-9 < 10)	37 (86.0%)	127 (78.4%)	164 (80.0%)
Yes (PHQ-9 ≥ 10)	5 (11.6%)	25 (15.4%)	30 (14.6%)
PC-PTSD score			
Mean (SD)	0.308 (0.893)	0.414 (0.967)	0.391 (0.950)
Median [Min, Max]	0 [0, 4.00]	0 [0, 4.00]	0 [0, 4.00]
Missing	4 (9.3%)	22 (13.6%)	26 (12.7%)
PTSD			
** No** (PC-PTSD-5 < 4)	38 (88.4%)	136 (84.0%)	174 (84.9%)
** Yes** (PC-PTSD-5 ≥ 4)	1 (2.3%)	4 (2.5%)	5 (2.4%)

### Patient’s perception of the status of their cancer and health professional opinion

According to the evaluation by HCPs, 113 had DC and 60 had PD. There was uncertainty in 32 cases. Thirty-three cases (16%) required a senior review, and consensus was reached. Fifteen cases had DC, ten had PD, and eight remained uncertain.

Comparing patients’ answers with the investigation of the clinical notes revealed a significant difference between the participants’ perception of their cancer status and the HCPs’ opinion (chi-squared test, p < 0.001). In particular, in the subgroup of participants whereby there was HCP-reported progressive disease, there was only 35.0% (n = 21/60) agreement. Notably, 36.7% (n = 22/60) believed their disease was under control and 28.3% (n = 17/60) were uncertain. Consistency in perception was highest amongst participants with DC according to the HCPs’ opinion, with 73.5% (n = 83/113). Among participants with uncertain disease status according to the HCPs’ opinion, 43.8% (n = 14/32) agreed, but 40.6% (n = 13/32) considered their disease under control and 15.6% (n = 5/32) believed that it was progressive (Table 4, supplementary Fig. 1).

**Table 4:** Patients’ understanding of Cancer Status and Health Professional Opinion in A. Overall population; B. Patients treated with curative intent; C. Patients treated with palliative intent

		Health Professional opinion			Sig
		DC	PD	Uncertain	
Patients’ understanding of cancer status	**A. Overall Population**				
	**DC**	83 (73.5%)	22 (36.7%)	13 (40.6%)	p < 0.001 (C)
	**PD**	7 (6.1%)	21 (35.0%)	5 (15.6%)	
	**Uncertain**	23 (20.4%)	17 (28.3%)	14 (43.8%)	
	**B. Patients treated with curative intent**				
	**DC**	23 (59.0%)	1 (50.0%)	1 (50.0%)	P = 0.999 (C)
	**PD**	1 (2.5%)	0 (0%)	0 (0%)	
	**Uncertain**	15 (38.5%)	1 (50.0%)	1 (50.0%)	
	**C. Patients treated with palliative intent**				
	**DC**	60 (81.1%)	21 (36.2%)	12 (40.0%)	P < 0.001 (C)
	**PD**	6 (8.1%)	21 (36.2%)	5 (16.7%)	
	**Uncertain**	8 (10.8%)	16 (27.6%)	13 (43.3%)	

Abbreviations: *DC *disease control, *PD* progressive disease, *c* chi-squared test

### Cancer perception by treatment intent

Analysis of the perception of treatment intent between participants and their responsible HCPs showed that there was no significant difference between the two parties in the group of patients receiving treatment with curative intent (adjuvant/neoadjuvant), (Table 4, Supplementary Fig. 2). In contrast, significant differences were observed between participants’ perceptions of their cancer status and HCPs’ opinions for patients receiving palliative care (chi-squared test p < 0.001, Table 4, supplementary Fig. 3). Among participants receiving palliative treatment with DC as assessed by their HCPs, 81.1% (n = 60/74) had a congruent understanding of their condition. However, only 36.2% (n = 21/58) of participants receiving palliative care with progressive disease shared this view, while 27.3% were unsure (n = 16/58) and 36.2% (n = 21/58) thought their disease was under control. Among patients with ambiguous disease status as assessed by HCPs, 43.3% (n = 13/30) expressed uncertainty, while 40.0% (n = 12/30) thought their condition was under control and 16.7% (n = 5/30) considered that they had disease progression.

### Association between demographic and psychosocial health outcomes with concordance status

We also examined whether there were differences in demographic, social and psychological health and wellbeing, as assessed using psychometric tools (GAD-7, PHQ-9, PTSD and WHO-5), between patients whose perception of their cancer status aligned with that of their healthcare professional (concordant), and those whose understanding differed (non-concordant) (Supplementary Table 1). The analysis revealed no statistically significant associations between concordance and the following demographic factors: gender (p = 0.595); age group (p = 0.406); marital status (p = 0.672); having children (p = 1.000); and living conditions (p = 0.431). Similarly, there were no significant associations between concordance and psychological measures such as anxiety (p = 0.271), depression (p = 0.959), PTSD (p = 1.000) and well-being (p = 0.984).

### Psychological health outcomes and patients’ cancer perception

Finally, we investigated the association between patients’ characteristics, including demographics and living conditions, and their perception of their cancer status, with psychological health outcomes (GAD-7, PHQ-9, PTSD and WHO-5) (Supplementary Tables 2–5). Patients who believed their disease was progressing, or who were uncertain, were at a higher risk of depression (OR: 6.42, p = 0.001, and OR: 3.86, p = 0.009, respectively) (Supplementary Table 4). However, there was no correlation between patients’ perception of their cancer status and their well-being, anxiety, or PTSD (Supplementary Tables 2, 3 and 5).

## Discussion

The primary aim of this study was to examine participants’ perceptions in relation to HCPs’ opinions of actual disease status in a convenience sampling of patients with colorectal cancer. Our study revealed significant discrepancy between participants’ and HCPs’ perceptions, particularly among patients receiving treatment with palliative intent and had progressive disease. Here, there was only a 36% concordance in their understanding of their progressive disease status.

Existing evidence suggests that patients with advanced, terminal cancer have a limited understanding of their prognosis or life expectancy [17]. Similarly, more than 25% of patients with advanced cancer were not aware of the actual disease condition, while the accurate stage of the disease was not easily described in various studies [18, 19]. Siverdran et al. described the discordance to understand the concept of early cancer and curative disease but in their study included only a small number of patients with colorectal cancer [19].

Several factors may contribute to the differences in perceptions between patients and HCPs [10]. Effective clinician-patient communication is essential for managing expectations and enhancing patient satisfaction, especially for those with advanced disease [20]. Patients’ understanding of their health and their ability to comprehend negative news may be influenced by many cultural, psychological and medical factors; therefore, individuals’ needs must be considered when conveying medical information [10]. HCPs should be mindful of how patients perceive their disease status within the broader context of their lives, and information shared should be individualized to account for each patient’s unique perspective and emotional state [6, 8, 21–23]. However, the ability to tailor a communication style to the patient can be limited by time constraints during clinic visits, lack of continuity of care as patients see several different clinicians during their cancer journey, and high patient volumes. More could be done to use specialist nurses and support workers during or after appointments where patients have been updated on their cancer status, to provide a second layer of verification of patient understanding.

Strategies to support the most vulnerable group of patients with progressive disease should be appraised, alongside appropriate resource allocation. The role of clinical nurse specialists has been shown to be crucial in following up on discussions after consultations with clinicians, ensuring understanding and reducing the risk of misinterpretation as well as providing emotional support [24]. It may also be helpful to dedicate more time to cases where progressive disease is discussed, as using teaching-back communication techniques can increase patient understanding and engagement, improve health literacy, and reduce health disparities where literacy gaps might exist [25, 26]. Training in communication skills for HCPs starting from the early academic years is significant and needs to be revisited throughout the career [27, 28]. However, our study design does not allow us to determine which specific interventions would be most effective. There is still a need for intervention trials that explicitly test the effectiveness of various approaches to supporting patients with progressive disease.

Fallowfield et al. emphasized the necessity of ongoing education to enhance communication skills for clinicians treating cancer patients in their randomized trial over 20 years ago [29]. Since then, communication training has been integrated into continuing professional development, particularly for the UK oncology workforce. This study emphasises that, whatever the experience of a clinical group, resource allocation and clinical time must be carefully tailored to the patient, and the provision of written information and the involvement of family members, particularly in difficult discussions about palliative care and disease progression, should be strongly considered [30].

This is one of the very few studies to explore the relationship between patients’ perceptions of their cancer status and psychological distress, and, to our knowledge, the first to do so using validated psychometric tools, offering a novel insight not previously explored. As could be expected progressive disease and uncertainty about the actual disease status was significantly related with depression. These results align with previous reports where fear of progression is linked with depression [31], and illness uncertainty correlates with emotional distress and a decline in quality of life [32]. Notably, demographic and psychological characteristics were found to have no significant influence on the alignment between healthcare providers’ and patients’ comprehension of cancer status.

A limitation of this study was that only 8.3% of recruited patients were of non-white ethnicity. This study identified that non-white patients were statistically significantly more likely to self-report depression or anxiety compared to white patients. However, the study wasn’t adequately powered to assess ethnic differences in understanding of disease status. Growing evidence has shown that there are ethnic inequalities in cancer patients’ experiences, with non-white patients being less likely to receive an understandable explanation of treatment side effects [33] and less likely to have their prognosis addressed [34]. This may arise from cultural, linguistic and psychological factors, and more work must be done to understand how these impact patient’s understanding. Although the vast majority of participants in this group were native British speakers, further understanding of illness perceptions among ethnic minorities is needed. The study exclusively recruited colorectal patients, although other studies have reported similar results in other disease groups [2, 6].

The cross-sectional design of this study limits the ability to determine whether misperceptions arise from poor communication or other factors, such as emotional burden or past experiences. Therefore, causal interpretations should be made cautiously, and longitudinal research is needed to better understand these relationships over time.

Another limitation of the study is that it was conducted during the COVID-19 pandemic, a time when many consultations were conducted over the phone to reduce the risk of infection. Both clinicians and patients were likely to be wearing face masks throughout, which can hinder both verbal and non-verbal communication. This likely posed another communication barrier, however our study from the early stages of the pandemic, when restrictions were even more severe, found that remote consultations during this period met the needs of service users, although the level of understanding was not assessed [35]. Similar results from Desideri et al. indicated high levels of patient satisfaction and treatment compliance for complex radiation treatment [36]. Although the pandemic represents a confounding factor, that does not allow safe generalisability of our findings, it is likely that similar communication challenges would persist regardless of consultation format. Consequently, the pandemic context should not be regarded as a ‘worst-case scenario’, but rather as an environment that may have amplified pre-existing issues.

Future research should aim to clarify the mechanisms underlying discordance between patients and clinicians. Priorities should include examining whether technical language hinders patient comprehension, whether clinicians intentionally soften difficult information and how emotional distress affects patients’ understanding of clinical discussions. It will also be essential to identify when in the care pathway misunderstandings most often arise. Addressing these questions will inform the development of targeted interventions to reduce discordance and enhance communication quality.

## Conclusion

This study highlights a significant lack of understanding among clinicians and patients, particularly those receiving palliative care for advanced disease. Patient understanding is particularly important in this population, as they may be approaching a pivotal moment for advanced care planning and end-of-life dialogue. Effective communication is essential to enable patients to make informed choices about their care and to involve them in decision-making processes.

Further efforts are needed to understand the many patient factors involved and to implement a personalised approach to patient communication. More research is needed to understand the influence of demographic characteristics on mental health outcomes and to develop tailored interventions to improve patient well-being and empower patients to be active participants in their healthcare.

## Supplementary Information

Below is the link to the electronic supplementary material.

ESM 1 (DOCX 5.88 MB)

## Data Availability

The datasets used and/or analysed during the current study are available from the corresponding author on reasonable request.
