# Supplementary material for: Exploring perceptual disparities: A study on the level of understanding of colorectal cancer care among patients and healthcare professionals
Source: Int J Colorectal Dis. 2026 Jan 8;41(1):22. doi: 10.1007/s00384-025-05064-9 (PMC12783256; doi:10.1007/s00384-025-05064-9)
Supplement: Supplementary file 1 — (DOCX 5.88 MB) [file 384_2025_5064_MOESM1_ESM.docx]

# Supplementary Tables & Figures

Contents

[Supplementary Tables & Figures 1](#_Toc192448954)

[Supplementary Figure 1. Patients’ understanding of Cancer Status and Health Professional Opinion in the overall population 2](#_Toc192448955)

[Supplementary Figure 2. Patients’ understanding of Cancer Status and Health Professional Opinion in patients treated with curative intent 3](#_Toc192448956)

[Supplementary Figure 3. Patients’ understanding of Cancer Status and Health Professional Opinion in patients treated with palliative intent 4](#_Toc192448957)

[Supplementary Table 1. Association Between Demographic, Psychosocial Scores, and Concordance Status 5](#_Toc192448958)

[Supplementary Table 2. Association Between Patient Characteristics Wellbeing (WHO-5) 6](#_Toc192448959)

[Supplementary Table 3. Association Between Patient Characteristics and Anxiety (GAD-7). 7](#_Toc192448960)

[Supplementary Table 4. Association Between Patient Characteristics Wellbeing and Depression (PHQ9) 8](#_Toc192448961)

[Supplementary Table 5. Association Between Patient Characteristics Wellbeing and Post-traumatic stress. 9](#_Toc192448962)

## Supplementary Figure 1. Patients’ understanding of Cancer Status and Health Professional Opinion in the overall population

| 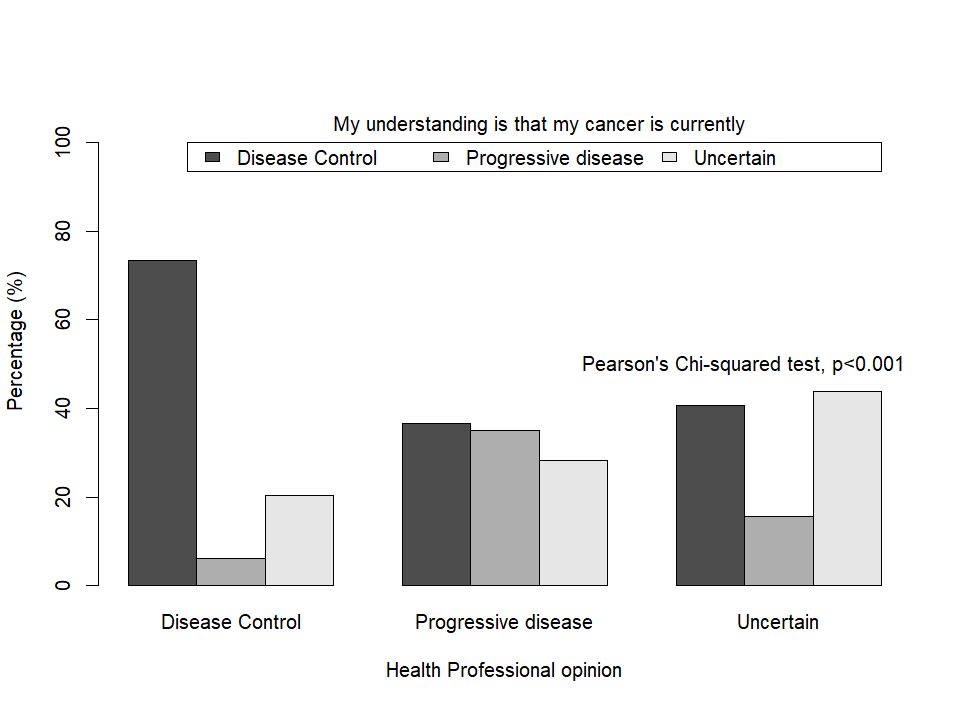 |
| --- |
| Supplementary Figure 1. Cancer Perception and Health Professional Opinion |

## Supplementary Figure 2. Patients’ understanding of Cancer Status and Health Professional Opinion in patients treated with curative intent

| 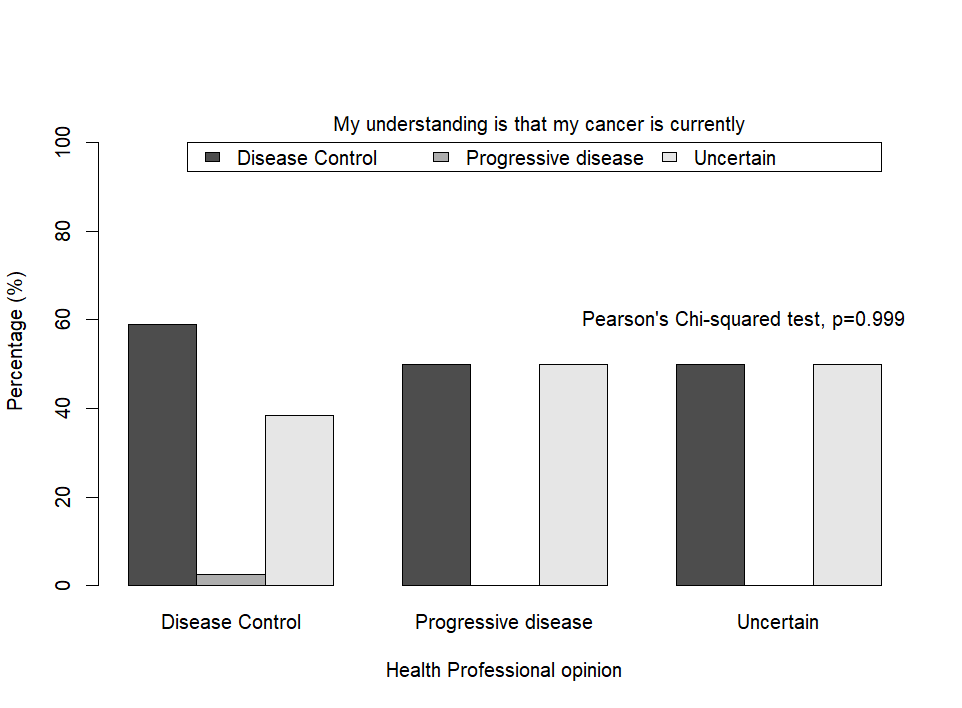 |
| --- |
| Supplementary Figure 2. Patients’ understanding of Cancer Status and Health Professional Opinion in patients treated with curative intent |

## Supplementary Figure 3. Patients’ understanding of Cancer Status and Health Professional Opinion in patients treated with palliative intent

| 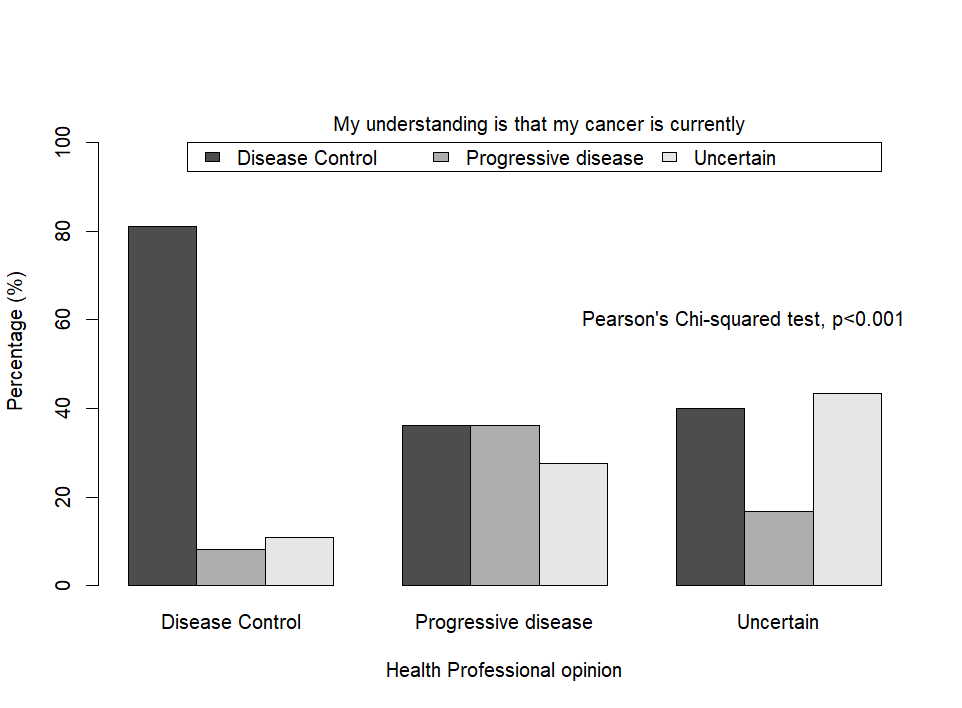 |
| --- |
| Supplementary Figure 3. Patients’ understanding of Cancer Status and Health Professional Opinion in patients treated with palliative intent. |

## Supplementary Table 1. Association Between Demographic, Psychosocial Scores, and Concordance Status

|  | **Concordance** | | |
| --- | --- | --- | --- |
|  | **No** | **Yes** | **P-value** |
| **Gender** |  | | |
| Male | 53 (44.5%) | 66 (55.5%) | 0.595 ^(C)^ |
| Female | 33 (39.8%) | 50 (60.2%) |  |
| **Age group** |  | | |
| ≥67 | 39 (39.0%) | 61 (61.0%) | 0.406 ^(C)^ |
| <67 | 48 (45.7%) | 57 (54.3%) |  |
| **Marital status** |  | | |
| Single/Divorced/Separated/Widowed | 29 (45.3%) | 35 (54.7%) | 0.672 ^(C)^ |
| In a relationship/Married/In civil partnership | 57 (41.0%) | 82 (59.0%) |  |
| **Do you have children?** |  | | |
| No | 19 (43.2%) | 25 (56.8%) | 1.000 ^(C)^ |
| Yes | 68 (42.2%) | 93 (57.8%) |  |
| **Living condition** |  | | |
| With Company | 65 (40.9%) | 94 (59.1%) | 0.431 ^(C)^ |
| Alone | 22 (48.9%) | 23 (51.1%) |  |
| **Anxiety (GAD7)** |  | | |
| No (GAD-7 <5) | 63 (43.8%) | 81 (56.3%) | 0.271 ^(C)^ |
| Yes (GAD-7 ≥ 5) | 16 (33.3%) | 32 (66.7%) |  |
| **Depression (PHQ-9)** |  | | |
| No (PHQ-9 < 10) | 67 (40.9%) | 97 (59.1%) | 0.959 ^(C)^ |
| Yes (PHQ-9 ≥10) | 13 (43.3%) | 17 (56.7%) |  |
| **PTSD** |  | | |
| No (PC-PTSD-5 < 4) | 73 (42.0%) | 101 (58.0%) | 1.000 ^(F)^ |
| Yes (PC-PTSD-5 ≥ 4) | 2 (40.0%) | 3 (60.0%) |  |
| **Wellbeing (WHO-5)** |  | | |
| Good (WHO-5 ≥ 50) | 58 (42.0%) | 80 (58.0%) | 0.984 ^(C)^ |
| Poor (WHO-5 <50) | 29 (43.3%) | 38 (56.7%) |  |
| Supplementary Table 1. Association Between Demographic, Psychosocial Scores, and Concordance Status  Abbreviations: (C): Chi-Square test; (F): Fisher’s exact test | | | |

## Supplementary Table 2. Association Between Patient Characteristics and Wellbeing (WHO-5)

| Wellbeing (WHO-5) |  | Good  well-being  (WHO-5 ≥ 50) | Poor  well-being  (WHO-5 <50) | Univariable analysis  OR with 95% CI | Multivariable analysis  OR with 95% CI |
| --- | --- | --- | --- | --- | --- |
| My understanding is that my cancer is currently | Disease control | 85 (72.0) | 33 (28.0) | - | - |
|  | Progressive disease | 20 (60.6) | 13 (39.4) | 1.67 (0.74-3.73, p=0.210) | 1.54 (0.66-3.50, p=0.310) |
|  | Uncertain | 33 (61.1) | 21 (38.9) | 1.64 (0.83-3.23, p=0.154) | 1.49 (0.73-3.03, p=0.268) |
| Age | Mean (SD) | 65.4 (10.3) | 65.3 (10.3) | 1.00 (0.97-1.03, p=0.967) | 1.00 (0.97-1.03, p=0.976) |
| Gender | Male | 87 (73.1) | 32 (26.9) | - | - |
|  | Female | 50 (60.2) | 33 (39.8) | 1.79 (0.99-3.27, p=0.055) | 1.62 (0.85-3.08, p=0.141) |
| Ethnicity | White/White British | 130 (69.1) | 58 (30.9) | - | - |
|  | Other | 8 (53.3) | 7 (46.7) | 1.96 (0.66-5.72, p=0.213) | 1.93 (0.62-5.90, p=0.246) |
| Marital status | Single/Divorced/Separated/Widowed | 42 (65.6) | 22 (34.4) | - | - |
|  | In a relationship/Married/In civil partnership | 96 (69.1) | 43 (30.9) | 0.86 (0.46-1.62, p=0.626) | 0.97 (0.38-2.68, p=0.956) |
| Do you have children | No | 32 (72.7) | 12 (27.3) | - | - |
|  | Yes | 106 (65.8) | 55 (34.2) | 1.38 (0.67-2.99, p=0.389) | 1.62 (0.70-4.03, p=0.276) |
| Live Alone | No | 108 (67.9) | 51 (32.1) | - | - |
|  | Yes | 30 (66.7) | 15 (33.3) | 1.06 (0.51-2.12, p=0.873) | 1.15 (0.38-3.56, p=0.802) |
|  | | | | | |

## Supplementary Table 3. Association Between Patient Characteristics and Anxiety (GAD-7).

| Dependent GAD7-score |  | No Anxiety  (GAD-7 <5) | Anxiety  (GAD-7 ≥ 5) | Univariable analysis  OR with 95% CI | Multivariable analysis  OR with 95% CI |
| --- | --- | --- | --- | --- | --- |
| My understanding is that my cancer is currently | Disease control | 90 (80.4) | 22 (19.6) | - | - |
|  | Progressive disease | 22 (68.8) | 10 (31.2) | 1.86 (0.75-4.43, p=0.168) | 1.89 (0.74-4.69, p=0.173) |
|  | Uncertain | 32 (66.7) | 16 (33.3) | 2.05 (0.95-4.37, p=0.065) | 2.13 (0.95-4.76, p=0.065) |
| Age | Mean (SD) | 66.2 (10.2) | 61.8 (10.7) | **0.96 (0.93-0.99, p=0.013)** | **0.96 (0.93-1.00, p=0.032)** |
| Gender | Male | 86 (77.5) | 25 (22.5) | - | - |
|  | Female | 56 (71.8) | 22 (28.2) | 1.35 (0.69-2.63, p=0.374) | 1.18 (0.57-2.44, p=0.645) |
| Ethnicity | White/White British | 135 (77.1) | 40 (22.9) | - | - |
|  | Other | 8 (53.3) | 7 (46.7) | **2.95 (0.98-8.73, p=0.048)** | 2.37 (0.75-7.27, p=0.130) |
| Marital status | Single/Divorced/Separated/Widowed | 47 (78.3) | 13 (21.7) | - | - |
|  | In a relationship/Married/In civil partnership | 96 (73.8) | 34 (26.2) | 1.28 (0.63-2.72, p=0.506) | 1.21 (0.42-3.86, p=0.737) |
| Do you have children | No | 31 (75.6) | 10 (24.4) | - | - |
|  | Yes | 113 (74.8) | 38 (25.2) | 1.04 (0.48-2.42, p=0.919) | 1.23 (0.49-3.30, p=0.666) |
| Live Alone | No | 111 (74.0) | 39 (26.0) | - | - |
|  | Yes | 32 (78.0) | 9 (22.0) | 0.80 (0.33-1.77, p=0.597) | 1.04 (0.30-3.77, p=0.945) |
| Supplementary Table 3. Association Between Patient Characteristics and Anxiety (GAD-7). | | | | | |

## Supplementary Table 4. Association Between Patient Characteristics and Depression (PHQ9)

| Depression (PHQ9) |  | No depression  (PHQ-9 < 10) | Depression  (PHQ-9 ≥10) | Univariable analysis  OR with 95% CI | Multivariable analysis  OR with 95% CI |
| --- | --- | --- | --- | --- | --- |
| My understanding is that my cancer is currently | Disease control | 102 (92.7) | 8 (7.3) | - | - |
|  | Progressive disease | 21 (65.6) | 11 (34.4) | **6.68 (2.42-19.26, p<0.001)** | **6.42 (2.23-19.43, p=0.001)** |
|  | Uncertain | 41 (78.8) | 11 (21.2) | **3.42 (1.29-9.43, p=0.014)** | **3.86 (1.41-11.05, p=0.009)** |
| Age | Mean (SD) | 65.3 (10.2) | 64.0 (10.6) | 0.99 (0.95-1.03, p=0.531) | 0.99 (0.95-1.03, p=0.517) |
| Gender | Male | 95 (84.8) | 17 (15.2) | - | - |
|  | Female | 66 (83.5) | 13 (16.5) | 1.10 (0.49-2.41, p=0.811) | 0.91 (0.36-2.24, p=0.843) |
| Ethnicity | White/White British | 154 (86.5) | 24 (13.5) | - | - |
|  | Other | 8 (57.1) | 6 (42.9) | **4.81 (1.47-15.08, p=0.007)** | **4.43 (1.22-15.75, p=0.020)** |
| Marital status | Single/Divorced/Separated/Widowed | 49 (81.7) | 11 (18.3) | - | - |
|  | In a relationship/Married/In civil partnership | 113 (85.6) | 19 (14.4) | 0.75 (0.34-1.74, p=0.487) | 0.72 (0.22-2.70, p=0.606) |
| Do you have children | No | 36 (85.7) | 6 (14.3) | - | - |
|  | Yes | 128 (84.2) | 24 (15.8) | 1.12 (0.45-3.22, p=0.812) | 1.07 (0.36-3.59, p=0.902) |
| Live Alone | No | 129 (84.9) | 23 (15.1) | - | - |
|  | Yes | 34 (82.9) | 7 (17.1) | 1.15 (0.43-2.80, p=0.761) | 0.82 (0.19-3.62, p=0.792) |
| Supplementary Table 4. Association Between Patient Characteristics Wellbeing and Depression (PHQ9). | | | | | |

## Supplementary Table 5. Association Between Patient Characteristics and Post-traumatic stress.

| PTSD-score |  | No PTSD  (PTSD-5 < 4) | PTSD  (PTSD-5 ≥ 4) | Univariable analysis  OR with 95% CI | Multivariable analysis  OR with 95% CI |
| --- | --- | --- | --- | --- | --- |
| My understanding is that my cancer is currently | Disease control | 101 (97.1) | 3 (2.9) | - | - |
|  | Progressive disease | 30 (100.0) | 0 (0.0) | p=0.993 | p=0.997 |
|  | Uncertain | 43 (95.6) | 2 (4.4) | 1.57 (0.20-9.77, p=0.630) | 2.39 (0.26-22.53, p=0.417) |
| Age | Mean (SD) | 65.7 (10.1) | 55.2 (8.9) | **0.92 (0.85-0.99, p=0.033)** | 0.93 (0.83-1.02, p=0.150) |
| Gender | Male | 99 (99.0) | 1 (1.0) | - | - |
|  | Female | 73 (94.8) | 4 (5.2) | 5.42 (0.78-107.31, p=0.134) | 3.86 (0.45-83.38, p=0.264) |
| Ethnicity | White/White British | 162 (97.6) | 4 (2.4) | - | - |
|  | Other | 11 (91.7) | 1 (8.3) | 3.68 (0.18-27.71, p=0.261) | 3.05 (0.13-32.75, p=0.391) |
| Marital status | Single/Divorced/Separated/Widowed | 58 (98.3) | 1 (1.7) | - | - |
|  | In a relationship/Married/In civil partnership | 115 (96.6) | 4 (3.4) | 2.02 (0.29-39.95, p=0.534) | 0.37 (0.03-8.66, p=0.440) |
| Do you have children | No | 38 (97.4) | 1 (2.6) | - | - |
|  | Yes | 136 (97.1) | 4 (2.9) | 1.12 (0.16-22.21, p=0.922) | 1.06 (0.11-26.25, p=0.963) |
| Live Alone | No | 133 (96.4) | 5 (3.6) | - | - |
|  | Yes | 41 (100.0) | 0 (0.0) | p=0.995 | p=0.996 |
| Supplementary Table 5. Association Between Patient Characteristics Wellbeing and Post-traumatic stress. | | | | | |
